# A role for G-CSF and GM-CSF in nonmyeloid cancers

**DOI:** 10.1002/cam4.239

**Published:** 2014-04-02

**Authors:** Alexander M Aliper, Victoria P Frieden-Korovkina, Anton Buzdin, Sergey A Roumiantsev, Alex Zhavoronkov

**Affiliations:** 1 Federal Clinical Research Center of Pediatric Hematology, Oncology and ImmunologySamory Mashela 1, Moscow, 117198, Russia; 2 HiBiotechnology, LLCPOB 522, Wellman, Iowa City, 52242, Iowa; 3 Shemyakin-Ovchinnikov Institute of Bioorganic ChemistryMiklukho-Maklaya 16/10, Moscow, 117997, Russia; 4 First Oncology Research and Advisory Center, LLCFersmana 11, Moscow, 117312, Russia; 5 Pirogov Russian National Research Medical UniversityOstrovitianov str. 1, Moscow, 117997, Russia; 6 Moscow Institute of Physics and TechnologyInstitutskiy lane 9, Dolgoprudny city, Moscow, 141700, Russian; 7 The Biogerontology Research Foundation, BGRF4 Hill Street, London, W1J 5NE, UK

**Keywords:** Bladder, bone, cancer, colorectal, G-CSF, glioma, GM-SF, lung, melanoma, metastasis, prostate

## Abstract

Granulocyte colony-stimulating factor (G-CSF) and granulocyte-macrophage colony-stimulating factor (GM-CSF) modulate progression of certain solid tumors. The G-CSF- or GM-CSF-secreting cancers, albeit not very common are, however, among the most rapidly advancing ones due to a cytokine-mediated immune suppression and angiogenesis. Similarly, de novo angiogenesis and vasculogenesis may complicate adjuvant use of recombinant G-CSF or GM-CSF thus possibly contributing to a cancer relapse. Rapid diagnostic tools to differentiate G-CSF- or GM-CSF-secreting cancers are not well developed therefore hindering efforts to individualize treatments for these patients. Given an increasing utilization of adjuvant G-/GM-CSF in cancer therapy, we aimed to summarize recent studies exploring their roles in pathophysiology of solid tumors and to provide insights into some complexities of their therapeutic applications.

## Introduction

Granulocyte and granulocyte-macrophage colony-stimulating factors (G-CSF and GM-CSF, respectively) regulate maturation of progenitor cells in the bone marrow into differentiated granulocytes, macrophages, and the T cells. In clinical oncology, recombinant G- or GM-CSFs are routinely used to correct neutropenia subsequent to chemotherapy and radiation. However, adjuvant G-/GM-CSF treatments have been suggested to occasionally enable tumor growth. Such adverse treatment outcomes are thought to occur due to certain solid tumors being addicted to G-/GM-CSF-dependent signaling by expressing endogenous cytokines and/or their cognate receptors (G-/GM-CSFR). Clinical case reports reveal that newly diagnosed patients with G-/GM-CSF(R)-positive tumors present with an advanced often metastatic disease suggesting an accelerated progression of such cancers. The putative mechanisms of progression bear similarities to those observed with an adjuvant use of recombinant cytokines, that is, induction of immune tolerance and angiogenesis. It is therefore an imperative to explore roles of G-CSF and GM-CSF in cancer in order to improve treatment outcomes and to better define eligible patients' cohorts. In this review, we aimed to present recent advances in studies addressing putative mechanisms and therapeutic uses of G-CSF and GM-CSF in several cancers of a nonmyeloid origin.

## Molecular mechanisms of G-CSF and GM-CSF signaling

### G-CSF and GM-CSF receptor–ligand complexes

**Figure 1:**
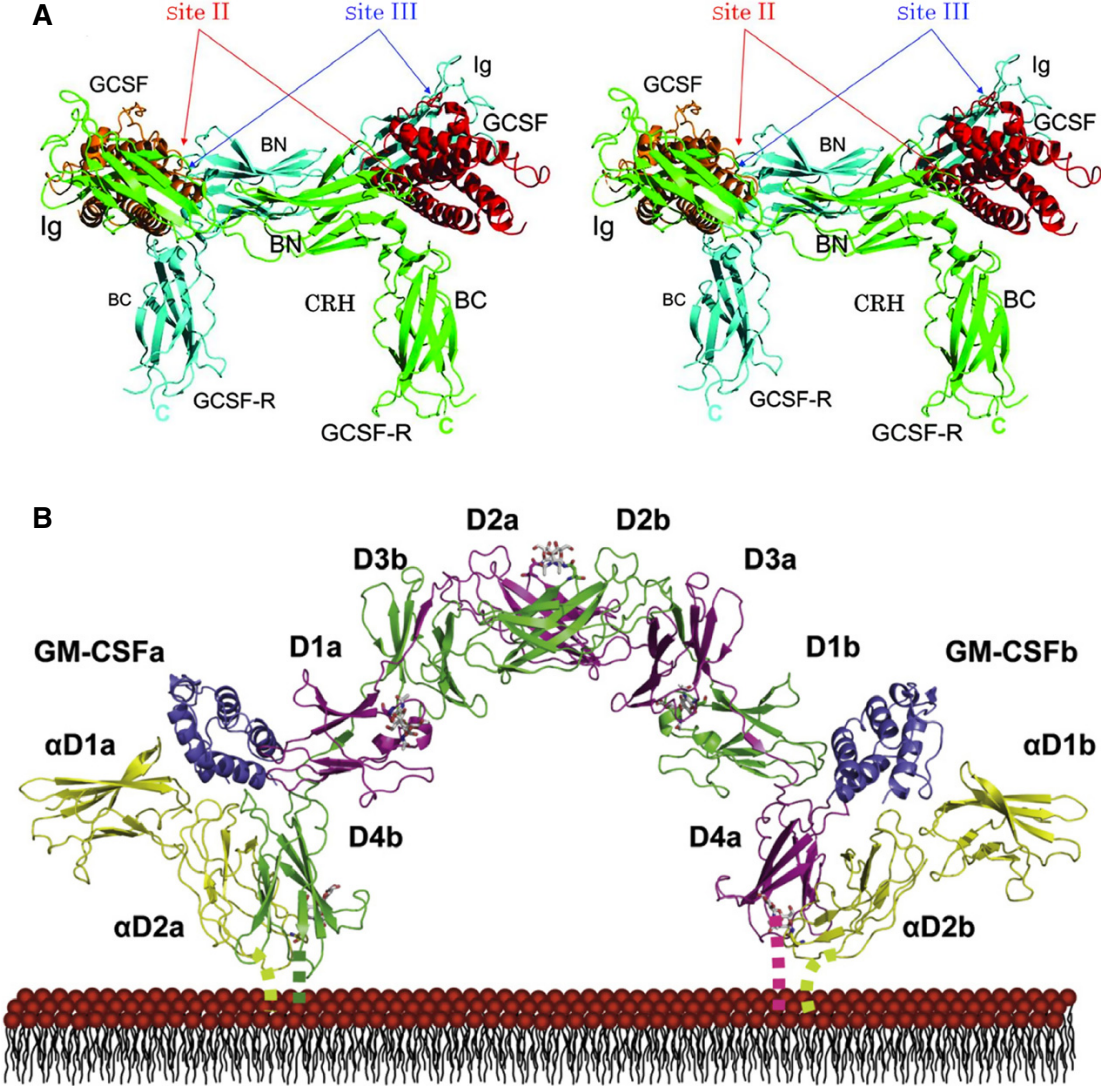
(A) The G-GSF receptor monomers (*green and blue*) consist of an extracellular Ig-like domain (*Ig*), a cytokine receptor homologous (*CRH*) domain, and three fibronectin-type III-like domains followed by a transmembrane region and a cytoplasmic domain. Upon the G-CSF (*red*) binding, receptors polymerize via Ig-like domains to form an active signaling complex. (Modified from 6). (B) The GM-CSF receptor is a complex of two *α*-subunits (*yellow*) and the two *β*c-subunits (*maroon and green*). The *α*-subunits ensure specificity of GM-CSF (*purple*) binding, whereas *β*c-subunits which are shared among GM-CSFR, IL-3R, and IL-5R provide high-affinity binding sites. The GM-CSFR localize extracellularly with domains of both *α*- and *β*c-subunits tethering them to the cell membranes (Modified from 7).

The G-CSF and GM-CSF are glycoproteins with molecular weights of 30 kDa and 22 kDa, respectively, secreted by the cells of the immune system, fibroblasts, and endothelium. They function to stimulate granulopoiesis, the innate immunity, and the differentiation of neural progenitor cells 1–5. Both cytokines require presence of their specific receptors to initiate intracellular signaling. Crystallographic studies depict activated G-CSFRs as tetramers where two receptor–ligand dimers form a complex on plasma membranes via Ig-like domains (Fig.1A) 6. In contrast, an activated GM-CSFR is a hexamer consisting of two ligand-selective *α*-subunits and the two nonselective *β*c subunits; each *α*-/*β*c-subunits dimer is thought to bind one ligand molecule (Fig.1B) 7–9. Moreover, activation of downstream signaling requires the two hexamers to form a dodecameric complex, a feature is thought to be unique to a GM-CSFR 7.

### Signal transduction pathways

**Figure 2:**
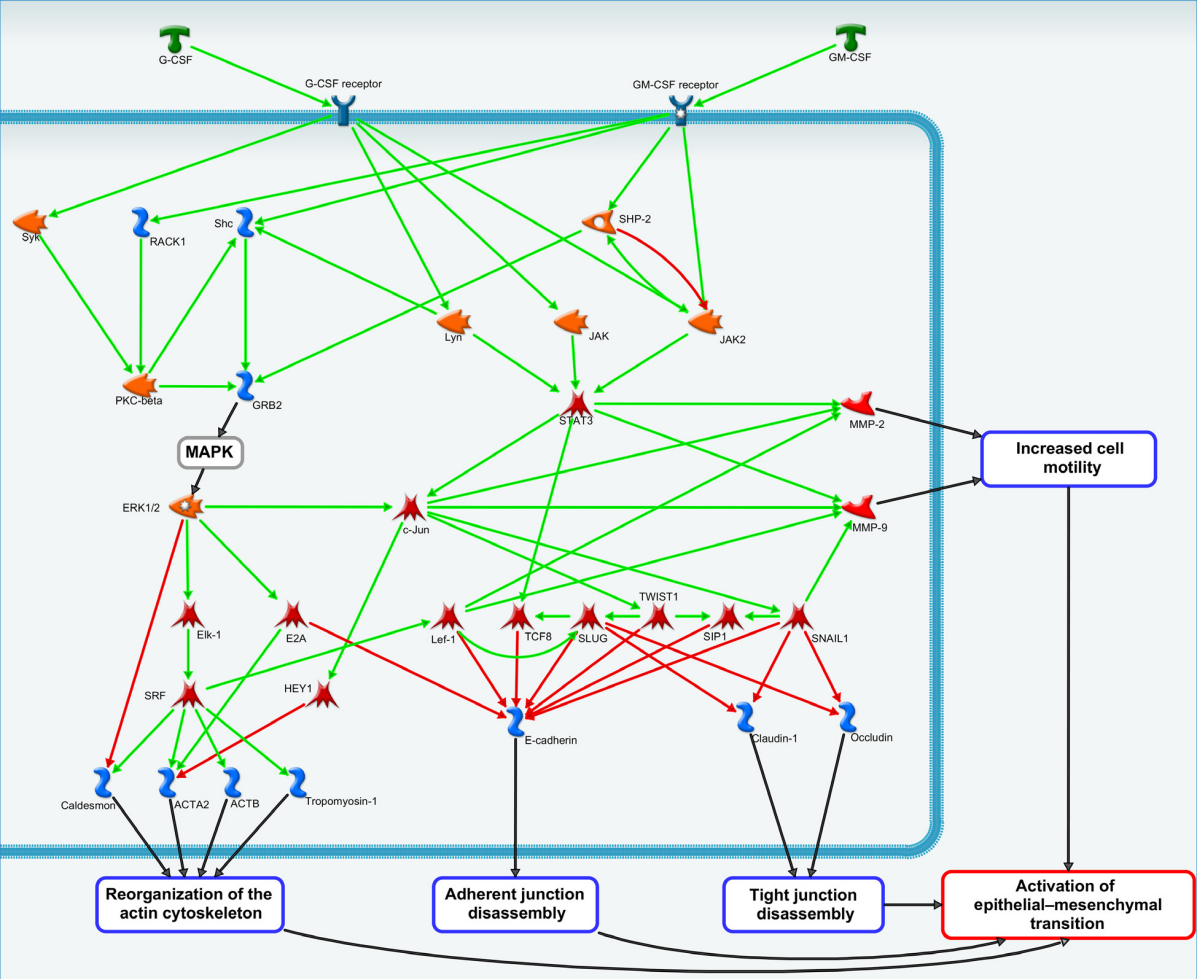
MetaCore™ pathways analysis has been utilized to generate a map of putative signal transduction pathways for G-CSF or GM-CSF to regulate epithelial to mesenchymal transition in cancer. The green arrows indicate activated signaling pathways, whereas red arrows depict inhibited pathways.

In physiological conditions, for example, during maturation of granulocyte/macrophage precursors, activated G-/GM-CSFR elicit phosphorylation of JAK kinases and subsequent recruitment of STAT5 transcription factor to effect cellular differentiation 7,10,11. G-CSF has also been shown to convey neuroprotection to central neurons upon increases in phosphorylation of PI3K/Akt pathway 7,10,11. In cancers of nonmyeloid origin, however, the G-/GM-CSF signaling cascades are less known. Highly annotated automatic pathways analysis tools, such as MetaCore™ (Thompson Reuters, New York) therefore may become indispensable in predicting such novel cascades. For example, Figure2 outlines a map of putative signal transduction pathways whereby G-CSF or GM-CSF regulate epithelial to mesenchymal transition (EMT), a crucial event in malignant transformation. The map suggests that a recruitment of *c-jun* proto-oncogene may occur downstream from Lyn/JAK/STAT3 or, alternatively, MAPK/ERK1/2 pathways upon their activation by cytokines. Recruitment of *c-jun* would promote reorganization of actin cytoskeleton, secretion of matrix metalloproteases, and a loss of cell to cell contact to increase cell motility and hence to facilitate dissemination. Indeed, G-/GM-CSF-dependent phosphorylation of JAK2 and recruitment of STAT-3 have previously been reported as required steps in tumor angiogenesis and vascularization 12,13. Moreover, signaling cascades regulating EMT have been shown to convey stem cell phenotype to neoplastic cells which would account for their ability to metastasize and for their multidrug resistance 14. To date, the contributions of G-/GM-CSF to cancer stem cell phenotypes are not clearly defined 15. It is noteworthy therefore that the MetaCore™ analysis proposes a role for G-/GM-CSF in maintaining a pool of stem cells in solid cancers via *c-jun-*dependent activation of SLUG, SNAIL1, or TWIST-1 transcription factors 16,17. Given a propensity of G-/GM-CSF(R)-expressing cancers toward accelerated growth and an early dissemination, it is feasible to predict that the experimental evidence will emerge as to their roles in sustaining a cancer stem cell phenotype.

## Iatrogenic induction of G-/GM-CSF-dependent tumor growth

Clinical reports and animal cancer models attest to the chemo- and/or antiangiogenic therapies or a radiation treatment inadvertently promoting cancer progression in part via G-CSF. Shaked et al. demonstrate a G-CSF-dependent mobilization of endothelial progenitor cells and a tumor regrowth in murine models of melanoma and a lung cancer following treatments with vascular disrupting agents (VDA) 18. Similar increases in plasma G-CSF levels have been detected in patients with solid tumors receiving VDA 19. Remarkably, G-CSF fails to mobilize endothelial progenitor cells in mice not bearing tumors suggesting modulatory effects of tumor microenvironment on bone marrow responses to cytokines 20. Gr1+CD11b+ myeloid-derived suppressor cells (MDSC) recruited to the tumor site may in part mediate these effects 21. It has also been proposed that G-CSF, but not GM-CSF, expression with simultaneous infiltration of Gr1+CD11b+ MDSC render tumors refractory to the subsequent antiangiogenic treatments 21. In murine models of pancreatic adenocarcinoma expressing RAS oncogene, the G-CSF-mediated resistance to antivascular endothelial growth factor (VEGF) therapies occurs through activation of RAS/MEK/ERK pathways and an Ets-induced overexpression of G-CSF 22. Furthermore, studies suggest that by recruiting MDSC, G-CSF induces a VEGF-independent angiogenesis in addition to increasing resistance to anti-VEGF drugs 22. It becomes apparent therefore that correction of cancer therapy-related neutropenia using recombinant G-/GM-CSF carries risks of disease recurrence thus necessitating stricter eligibility criteria for patients requiring these treatments.

## Tumors secreting G-/GM-CSF

### Lung cancer

Primary and metastatic lung cancers are by far the most frequently occurring type of malignancy driven by the ectopically secreted G-/GM-CSF 23–26. Different histopathological types of a non-small-cell lung cancer (NSCLC) comprise a majority of cytokine-producing lung tumors although a case of G-CSF-secreting lung sarcoma has also been described 27,28. At a time of diagnosis, patients present with an advanced disease and a profound paraneoplastic leukocytosis. Elevated G-CSF levels have been proposed as a marker of shorter survival in NSCLC patients even if a subsequent resection of a cytokine-secreting tumor has been successful 24,29.

Various cell types have been proposed as putative sources of G-/GM-CSF in lung cancers. Specifically, tumor-associated endothelial cells may secrete cytokines and thus promote angiogenesis and metastasis via increases in expression of cell adhesion molecules 30. In addition, studies in animal models propose such function for Gr1+CD11b+ MDSC 31. Surprisingly, microarray data reveal augmentations of a GM-CSF gene in small-cell lung cancers but not in NSCLC possibly suggesting posttranscriptional mechanisms for an increased secretion of this cytokine 32. Given that G-/GM-CSF may accelerate progression and distant metastases in lung cancers, caution is warranted when using recombinant cytokines as an adjuvant treatment in these patients 33.

### Glioma

Glioma is the most common type of a primary malignant brain tumor in adults with universally poor prognosis. Gliomas more often than other G-/GM-CSF-secreting cancers also express intratumoral cognate receptors; augmented G-/GM-CSF(R) levels have been found to correlate with higher tumor grade 34–36. In these cancers, the G-/GM-CSF(R) promote progression possibly using auto-/paracrine activation of antiapoptotic and pro-angiogenic pathways via activation of STAT-3 transcription factor or an increased expression of VEGF/VEGFR 37–41. The origins of cytokine- secreting cells in glioma are not completely known. For example, tumor cells and tumor-associated microglia, but not mesenchymal stromal cells, have been proposed for this role 42–44. In experimental models of glioblastoma, decreasing G-/GM-CSF levels attenuate invasiveness and cell proliferation thus suggesting modulatory effects of these cytokines on tumor microenvironment 42. Consistent with these findings, accumulation of MDSC concomitant with increases in G-CSF levels has been shown in patients with glioma 45. Remarkably, a combination treatment consisting of a recombinant G-CSF and an interferon-gamma promotes maturation of tumor-associated dendritic cells 46,47. The induction of immune response by this treatment shows relative efficacy in prolonging survival in experimental gliomas 46,47. However, G-CSF monotherapy does not provide similar benefits which may be consistent with the aforementioned oncogenic roles of G-CSF 45–48. The lack of understanding of G-/GM-CSF roles in gliomas necessitates further research in pursuit of safer G-/GM-CSF-based therapies for patients with these cancers.

### Bladder cancer

A G-CSF was initially purified from the human bladder carcinoma cell line 5637 49 thus implying a role for this cytokine in progression of bladder malignancies. Tachibana et al. reported an autocrine growth induction after heterologous G-CSFR has been engineered into the G-CSF-secreting tumor cells from a resected bladder carcinoma 50. Growth potentiation may have possibly occurred via beta1-integrin-dependent augmentation of cell adhesion and invasion 50–52. In English scientific literature, clinical cases of bladder cancers secreting G-/GM-CSF or expressing their cognate receptors are not common 53–57. Patients present with an advanced disease and a marked leukocytosis in the absence of secondary infection or a myeloproliferative disorder 53–57. Peripheral blood smears show an abundance of differentiated neutrophils without left shift consistent with an ectopic induction of normal granulopoiesis 54. Responses to treatments are noticeably variable. Normalization of the white blood cell counts and cytokine levels upon successful resection of a tumor has been reported 53–57. Conversely, rapid metastatic spread and patient's demise despite therapeutic interventions has also been documented 53–57. Refractory cases are speculated to reflect possible involvement of endogenous tumor G-/GM-CSFRs 54. The microarray data report small but statistically significant increases in gene expression for both GM-CSF and *α*-subunits of GM-CSFR in bladder cancers compared to normal tissues 58,59. To the contrary, changes in G-CSF or its receptor gene expression have not been found 58,59. Given the rarity of G-/GM-CSF-secreting bladder tumors and an increasing use of recombinant G-/GM-CSF, appropriate diagnostic approaches are needed to identify patients to whom such therapies may be detrimental 60–62.

### Colorectal cancers

The elevated GM-CSF plasma levels have been found in certain patients with colorectal cancers thus implying this cytokine contributions to a disease progression 63. Gene expression arrays identified more than one third of human and murine colorectal cancers as secreting GM-CSF 64. Unlike in many other cancers, however, ectopic secretion of GM-CSF driven by demethylation of its gene promoter conveys antitumor effects that are both immune mediated and immune independent 64. The immune-independent effects require GM-CSFR which upon ligand binding significantly attenuates tumor formation 64. Moreover, patients with colorectal cancers whose tumors test positive for GM-CSF/GM-CSFR show improved overall 5-year survival 64. In contrast, clinical case reports of G-CSF-secreting cancers of colon and rectum describe patients presenting with large tumors and distal metastases 65,66. Despite surgical resection resulting in reduced G-CSF plasma levels, the overall survival of these patients is poor implying oncogenic functions for G-CSF 65,66. The aforementioned studies thus propose differential roles for GM-CSF and G-CSF in cancers of colon and rectum. This knowledge would undoubtedly translate into the individualized use of (neo)adjuvant cytokines depending on a molecular profile of a particular tumor.

### Melanoma and skin carcinoma

Melanoma is a rapidly progressing incurable skin cancer with a propensity to metastasize early due to immunosuppression and growth induction in part occurring via G-/GM-CSF. Clinical case studies report existence of are albeit severe G-CSF-secreting melanomas 67,68. That necessitates their consideration in a differential diagnosis given the use of adjuvant GM-CSF in these patients 67,68. Melanoma cells in vitro have been found to express G-CSFR transcript; increases in cell proliferation, however, do not occur upon G-CSF stimulation possibly reflecting an absence of a G-CSFR protein 69. In contrast, a role of GM-CSF in dissemination of melanoma is controversial. Studies using a murine model indicate that under hypoxic conditions tumor-associated macrophages upon stimulation with GM-CSF secrete a soluble VEGF receptor 70. The receptor inactivates VEGF and thus exerts antiangiogenic effects 70. A simultaneous stabilization of a hypoxia-induced transcription factor HIF-2*α* augments transcription of a VEGF receptor gene and enhances the antiangiogenic actions of GM-CSF in this model 70. In agreement with these observations, clinical trials of adjuvant GM-CSF monotherapy in patients with locally advanced melanoma demonstrate a decrease in the melanoma-specific deaths without improvements in a disease-free survival 71. However, other studies also utilizing murine models of melanoma have found positive correlations between increased GM-CSF levels and growth of lesions 72. Furthermore, a cytokine-dependent infiltration of tumors with Gr1+CD11+ MDSC consistent with tumorigenic effects of GM-CSF has also been documented 72. Controversies concerning the roles of G-/GM-CSF in melanoma may arise in part due to their complex synergistic inputs into a disease progression similar to those found in skin carcinoma 73. Authors of this study demonstrate that synergy between G-CSF and GM-CSF augments cell proliferation and invasiveness in addition to an early recruitment of tumor-associated macrophages to a tumor site 73. Thus, G-CSF in melanoma and skin carcinomas exacerbates disease progression due to pro-angiogenic and immunosuppressive actions. To the contrary, GM-CSF may demonstrate antitumor activity via modulating recruitment of tumor-associated macrophages and their VEGFR secretion.

### Bone metastases in cancers of prostate and breast

A role for G- or GM-CSF in cancers of prostate is less defined. Reports of G-/GM-CSF-secreting prostate tumors in English scientific literature are uncommon thus implying lesser significance of these cytokines for a prostate cancer progression. However, in vitro and animal models of this disease implicate G-/GM-CSF in promoting dissemination and bone metastasis. Namely, costimulation with G-CSF and a stem cell factor enhances cancer stem cell phenotype via upregulation of Oct3/4 transcription factor, NANOGP8 pseudogene and ABCG2 transporter 74. In murine xenograft models, GM-CSF has been found to facilitate metastatic seeding of prostate cancer cells in the bone by enhancing osteoclastic activity 75. Interestingly, this phenomenon has been observed while animals were undergoing a treatment with GM-CSF for a chemotherapy-induced leukopenia hence suggesting growth-promoting effects of a therapeutic GM-CSF 75. Similar exacerbation of an osteoclastic bone resorption has been reported in patients with breast cancer that metastasized to the bone 76. Specifically, in metastatic tissues the NF-kappaB transcription factor has been found to target GM-CSF gene to activate osteoclastogenesis and thus to facilitate homing of malignant cells in bone tissues. Given that cancer dissemination reflects a presence of cancer stem cells, their activation via administration of G-CSF has been proposed as a therapeutic strategy to augment their chemosensitivity 77. In this study, authors speculate that an increased relapse-free survival of breast cancer patients who received an adjuvant G-CSF is due to a diminished drug resistance of neoplastic cells populating bone micrometastases.

## Conclusions

Solid tumors of every origin present with many dissimilarities in their cellular composition and the molecular mechanisms of progression. Tumors addicted to G-/GM-CSF(R) signaling represents a very distinct molecular subset among other malignancies. For example, they utilize signaling pathways of normal hematopoiesis and the cytokine-mediated auto-/paracrine growth augmentation in addition to immune suppression. The aforementioned signaling modalities albeit not common in solid neoplasms contribute to some of the most advanced cases. Table1 briefly summarizes the pro- and antitumorigenic roles of G-/GM-CSF in cancers of different origins. Prompt diagnosis based on a tumor cytokine/receptor profile would aid in individualizing the anticancer treatment choices for these patients. In particular, stricter eligibility criteria for adjuvant use of G-/GM-CSF would prevent certain adverse effects, for example, exacerbation of tumor growth in cancers addicted to G-/GM-CSF.

**Table 1:** Summary of pro- and antitumorigenic roles of G-CSF and GM-CSF.

Tumor type	G-CSF	GM-CSF
NSCLC	Angiogenic, immunosuppressive via MDSC	
Glioma	Auto-/paracrine growth stimulation	
Bladder carcinoma	Auto-/paracrine growth stimulation	?
Colorectal cancer	Tumorigenic	Immune-mediated and immune-independent tumor suppression
Melanoma	Tumorigenic	Antiangiogenic via soluble VEGFR Tumorigenic via MDSC
Skin cancer	Synergistically tumorigenic and angiogenic	
Bone metastases	Sustain cancer stem cell phenotype	

G-CSF and GM-CSF differentially regulate tumor growth and metastasis in solid cancers. NSCLC, non-small-cell lung cancer; VEGFR, vascular endothelial growth factor receptor; MDSC, myeloid-derived suppressor cells; ?, no discernible evidence of an oncogenic or a tumor suppressor role has been found.
